# Optimization of Ribosome Structure and Function by rRNA Base Modification

**DOI:** 10.1371/journal.pone.0000174

**Published:** 2007-01-24

**Authors:** Jennifer L. Baxter-Roshek, Alexey N. Petrov, Jonathan D. Dinman

**Affiliations:** Department of Cell Biology and Molecular Genetics, University of Maryland, College Park, Maryland, United States of America; Victor Chang Cardiac Research Institute, Australia

## Abstract

**Background:**

Translating mRNA sequences into functional proteins is a fundamental process necessary for the viability of organisms throughout all kingdoms of life. The ribosome carries out this process with a delicate balance between speed and accuracy. This work investigates how ribosome structure and function are affected by rRNA base modification. The prevailing view is that rRNA base modifications serve to fine tune ribosome structure and function.

**Methodology/Principal Findings:**

To test this hypothesis, yeast strains deficient in rRNA modifications in the ribosomal peptidyltransferase center were monitored for changes in and translational fidelity. These studies revealed allele-specific sensitivity to translational inhibitors, changes in reading frame maintenance, nonsense suppression and aa-tRNA selection. Ribosomes isolated from two mutants with the most pronounced phenotypic changes had increased affinities for aa-tRNA, and surprisingly, increased rates of peptidyltransfer as monitored by the puromycin assay. rRNA chemical analyses of one of these mutants identified structural changes in five specific bases associated with the ribosomal A-site.

**Conclusions/Significance:**

Together, the data suggest that modification of these bases fine tune the structure of the A-site region of the large subunit so as to assure correct positioning of critical rRNA bases involved in aa-tRNA accommodation into the PTC, of the eEF-1A•aa-tRNA•GTP ternary complex with the GTPase associated center, and of the aa-tRNA in the A-site. These findings represent a direct demonstration in support of the prevailing hypothesis that rRNA modifications serve to optimize rRNA structure for production of accurate and efficient ribosomes.

## Introduction

Modification of ribonucleotides represents a way to expand the topological potentials of RNA molecules beyond those afforded by each of the four bases alone. Extensive research into rRNA modification has resulted in mapping of the majority of pseudouridine (*Ψ*) and 2′-O-methylation (Nm) residues in eukaryotic and archaeal ribosomes, and in identification of the snoRNA molecules that guide their modification. Despite this, little is understood about the functional roles of nucleotide modification. It is known that nucleotide modifications within the ribosome are not located randomly. This is most clearly seen in the ribosomal large subunit, where modifications cluster in highly conserved areas of the ribosome devoted to peptidyl transfer, sites of A- and P tRNA binding, the peptide exit tunnel and intersubunit bridges [1]–[4]. This clustering is conserved in organisms ranging from *E. coli* to humans with the number of modifications increasing with evolutionary complexity [5]. *In vitro* reconstituted *E. coli* ribosomes lacking rRNA modifications were severely defective in catalytic activity [6], and global disruption of *Ψ* or Nm formation *in vivo* resulted in strong growth defects in yeast [7], [8]. These essential modifications tend to be performed by snoRNPs that also harbor components essential for rRNA processing. However, most snoRNAs responsible for guiding rRNA modification can be individually deleted with minimal detriment to the organism [3], [9]. In fact, disruption/deletion of pseudouridine synthase proteins responsible for modification of only two or three residues in *E. coli* did not produce discernable differences in exponential growth rates between wild-type and mutant stains *in vivo*. However, rRNA modification mutants were strongly out competed by isogenic wild-type strains in competition experiments, suggesting a growth advantage conferred by the modifications [10], [11]. The prevailing hypothesis is that, although rRNA modifications are individually dispensable for survival, together they may serve to optimize rRNA structure for production of accurate and efficient ribosomes.

Based on the chemical properties of *Ψ* and Nm residues, their possible functional roles can be inferred but not established. It has been suggested that *Ψ* residues may contribute to RNA stability by altering potentials for base stacking, and by offering an extra hydrogen bond donor as compared to uridine [12], [13]. Nm residues offer protection against hydrolysis by bases and nucleases and can promote RNA structural changes by changing the hydration sphere around the 2′ oxygen, blocking sugar edge interactions and favoring the 3′endo ribose configuration [13], [14]. Thermodynamic and NMR based studies revealed that a *Ψ* residue can stabilize an RNA hairpin structure when located at a stem loop junction, and is slightly destabilizing when located in single-stranded loop regions [15]. Recent NMR studies of the highly conserved and highly modified large subunit rRNA (LSU) helix 69 of the human ribosome observed discernable but subtle secondary structure differences between rRNA with and without the modifications [16]. Functional and structural studies have shown that rRNA modification defects can impact on translation rates and ribosome integrity. In *E. coli*, mutants lacking methylation of the m^1^G745 residue located in the LSU exhibited decreased growth rates, decreased rates of polypeptide chain elongation, defects in ribosome profiles, and resistance to viomycin [17]. In yeast, knockout strains were made lacking each of six snoRNA genes that guide pseudouridylation of residues in the PTC of the ribosome, as well as one strain that lacked all six genes [18]. Only one individual mutant, the snR10 deletion strain, had phenotypic defects, but deletion of all six snoRNA genes promoted moderate defects in growth and translation rates, paromomycin hypersensitivity, and changes in ribosome profiles. *In vivo* DMS studies also revealed altered LSU rRNA structure for the multiple snoRNA deletion strain. Other functional studies have centered around two methylated nucleotides, mU_2920_ and mG_2921_, in the A loop of the yeast ribosome. There are two components thought to be involved in the methylation of these rRNA residues: the guide snoRNA snR52, and the site-specific methyltransferase Spb1p, an essential yeast nucleolar protein. Primer extension analysis revealed a functionally redundant pathway whereby snR52 or Spb1p could methylate residue Um_2920_ [19]. Later thin layer chromatography experiments revealed a different mechanism whereby Spb1p and snR52 were responsible for methylation of Gm_2921_ and Um_2920_ respectively, and showing that Spb1p could methylate residue Um_2920_ in the absence of snR52 [20]. Despite this discrepancy, it is clear that deleting both snR52 and Spb1p resulted in strong defects in growth rates, altered polysome profiles, and paromomycin hypersensitivity [19], making Spb1p an important exception to the snoRNA guided modification rule in eukaryotes. The *E. coli* homolog of Spb1p, FtsJ/RrmJ, methylates 23S rRNA residue Um_2552_ the equivalent of yeast Um_2920_ [21], and deletion of this protein in *E. coli* resulted in severe growth defects, temperature sensitivity, and altered ribosome profiles [22].

Despite their high level of conservation and distribution in functionally important areas of the ribosome, the functions of individual rRNA modifications belie their importance with a lack of defects in their absence. However, the changes in ribosome profiles and rRNA structures in multiple mutants suggest the intriguing possibility that they may each contribute to refining the structure and function of the translational apparatus. In order to more fully understand this, several strains harboring single deletion mutations, and one containing two gene deletions of previously characterized snoRNAs known to modify the PTC of the yeast ribosome were first characterized using a wide variety of genetic assays designed to assess translational fidelity. The results show that defects in rRNA modification produce allele specific mutant phenotypes including increased sensitivity to translational inhibitors; defects in virus propagation; changes in translational fidelity as monitored by +1 and −1 PRF, discrimination between cognate- and near-cognate aa-tRNAs, and recognition of termination codons. These analyses led to more detailed biochemical characterization of two mutants, demonstrating their increased affinities for aa-tRNAs and decreased rates of peptidyltransfer. rRNA chemical deprotection studies using a mutant defective in its ability to Nm Gm2921 and Um2920 identified structural changes in five positions. Specifically, at U2923 in the 25S rRNA A-loop in the peptidyltransferase center, at A2932 and A2933, which help to coordinate correct folding of the helix 90 – 92 structure, and at C2848 where the tip of helix 89 interacts with the GTPase-associated center, and at C2851, where helix 89 interacts with the T-stem of aa-tRNA. Together, the data suggest that modification of these bases fine tune the structure of the A-site region of the large subunit so as to assure correct positioning of critical rRNA bases involved in aa-tRNA accommodation into the PTC, of the eEF-1A•aa-tRNA•GTP ternary complex with the GTPase associated center, and of the aa-tRNA in the A-site. These findings represent the first direct demonstration in support of the prevailing hypothesis that rRNA modifications serve to optimize rRNA structure for production of accurate and efficient ribosomes.

## Results

**Figure 1:**
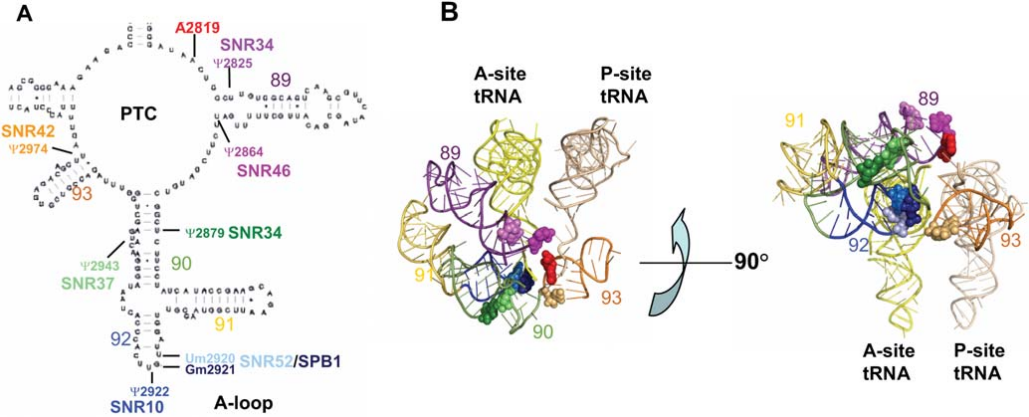
25S rRNA in the peptiptidyl transferase center of yeast. (A) Secondary structure of yeast 25S rRNA in the PTC. snoRNAs targeted for this study are indicated along with the residues they modify. *Ψ* – pseudouridylated residue; Nm – 2′-*O*-ribose methylated residue. Helices are numbered in black. (B) Three dimensional representation of the *E. coli* PTC [34]. Modified residues are labeled by the colors indicated in panel A. *Left*: view into the PTC from the top of the LSU, *righ*t: 90° rotation of *Left*. Helices and tRNAs are labeled.

In order to more precisely determine the role of rRNA modifications in the translational fidelity of the ribosome, yeast strains lacking several previously characterized snoRNAs and one protein that modify residues around the peptidyltransferase center of the ribosome were chosen for characterization. The strains contain single knockouts of the snoRNAs snR10, snR34, snR37, snR42, and snR46 which together pseudouridylate six rRNA residues in the PTC of the yeast ribosome, with snR34 modifying two of those residues. Single and double knockout strain snr52 and a methylase deficient mutant of the essential yeast protein Spb1, which are responsible for methylation of mG_2921_ and mU_2920_, were also used in this study. Since Spb1p is an essential yeast protein, a methylase deficient mutant with a D to A substitution affecting the AdoMet-binding site was used [19], [20]. Mutant strains *snr10* *Δ*, *spb1DA*, and *spb1DA*/*snr52* *Δ* have slow growth phenotypes. The locations of the modified bases modified are shown in figure 1.

### rRNA modification mutants show sensitivity to translation inhibitors

**Figure 2:**
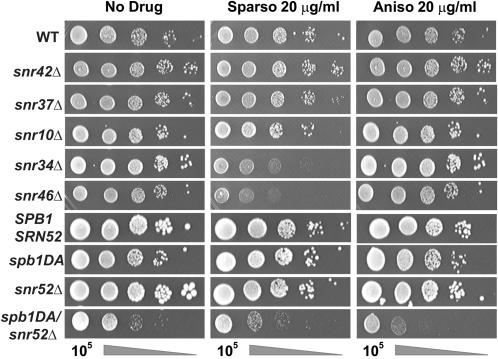
Sensitivity of rRNA base modification mutants to Translational Inhibitors. Mutant and isogenic wild-type yeast strains were spotted as ten fold dilutions from 10^5^ to 10^1^ CFU onto YPAD media containing 20 µg/ml anisomycin or sparsomycin. Cells were incubated for 3 days at 30°C, and growth was monitored as compared to growth on plates in the absence of drug. Each strain and drug was assayed at least twice.

Protein translation inhibitors that specifically interact with the ribosome provide sensitive and convenient probes for changes in ribosome function. Anisomycin, which binds the A-site crevice that normally accepts the amino acid side-chains of A-site bound aminoacyl-tRNAs [23] interfering with the binding of 3′ end of the aa-tRNA [24]–[26], was used to probe the A-site of the peptidyltransferase center (PTC). Sparsomycin, which binds on top of the CCA end of a P-site bound substrate and interacts with it interfering [27] with the binding of the 3′ end of the peptidyl-tRNA [28]–[30] was also used as a probe for functional changes in the PTC. Previous studies reported that *spb1DA* mutants were sensitive to paromomycin, and that sparsomycin had no effect [19]. Further, the *snr10* *Δ* mutant was also shown to be sensitive to paromomycin [18]. To obtain drug sensitivity profiles for all of the mutants, standard 10-fold dilution spot assays were performed in the presence of anisomycin or sparsomycin (20 µg/ml each). As shown in Figure 2 and summarized in Table 1, *spb1DA*/*snr52* *Δ* cells were anisomycin hypersensitive, and *snr34* *Δ* and *snr46* *Δ* strains were hypersensitive to sparsomycin.

**Table 1:**
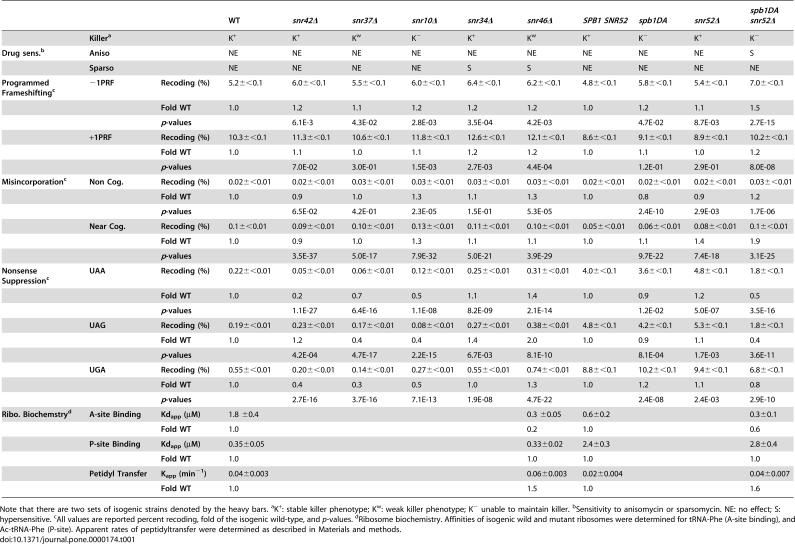
Summary of rRNA modification mutant phenotypes.

			WT	*snr42* *Δ*	*snr37* *Δ*	*snr10* *Δ*	*snr34* *Δ*	*snr46* *Δ*	*SPB1 SNR52*	*spb1DA*	*snr52* *Δ*	*spb1DA snr52* *Δ*
	**Killer^a^**		K^+^	K^+^	K^w^	K^−^	K^+^	K^w^	K^+^	K^−^	K^+^	K^−^
**Drug sens.^b^**	**Aniso**		NE	NE	NE	NE	NE	NE	NE	NE	NE	S
	**Sparso**		NE	NE	NE	NE	S	S	NE	NE	NE	NE
**Programmed Frameshifting^c^**	**−1PRF**	**Recoding (%)**	5.2±<0.1	6.0±<0.1	5.5±<0.1	6.0±<0.1	6.4±<0.1	6.2±<0.1	4.8±<0.1	5.8±<0.1	5.4±<0.1	7.0±<0.1
		**Fold WT**	1.0	1.2	1.1	1.2	1.2	1.2	1.0	1.2	1.1	1.5
		***p*** **-values**		6.1E-3	4.3E-02	2.8E-03	3.5E-04	4.2E-03		4.7E-02	8.7E-03	2.7E-15
	**+1PRF**	**Recoding (%)**	10.3±<0.1	11.3±<0.1	10.6±<0.1	11.8±<0.1	12.6±<0.1	12.1±<0.1	8.6±<0.1	9.1±<0.1	8.9±<0.1	10.2±<0.1
		**Fold WT**	1.0	1.1	1.0	1.1	1.2	1.2	1.0	1.1	1.0	1.2
		***p*** **-values**		7.0E-02	3.0E-01	1.5E-03	2.7E-03	4.4E-04		1.2E-01	2.9E-01	8.0E-08
**Misincorporation^c^**	**Non Cog.**	**Recoding (%)**	0.02±<0.01	0.02±<0.01	0.03±<0.01	0.03±<0.01	0.03±<0.01	0.03±<0.01	0.02±<0.01	0.02±<0.01	0.02±<0.01	0.03±<0.01
		**Fold WT**	1.0	0.9	1.0	1.3	1.1	1.3	1.0	0.8	0.9	1.2
		***p*** **-values**		6.5E-02	4.2E-01	2.3E-05	1.5E-01	5.3E-05		2.4E-10	2.9E-03	1.7E-06
	**Near Cog.**	**Recoding (%)**	0.1±<0.01	0.09±<0.01	0.10±<0.01	0.13±<0.01	0.11±<0.01	0.10±<0.01	0.05±<0.01	0.06±<0.01	0.08±<0.01	0.1±<0.01
		**Fold WT**	1.0	0.9	1.0	1.3	1.1	1.1	1.0	1.1	1.4	1.9
		***p*** **-values**		3.5E-37	5.0E-17	7.9E-32	5.0E-21	3.9E-29		9.7E-22	7.4E-18	3.1E-25
**Nonsense Suppression^c^**	**UAA**	**Recoding (%)**	0.22±<0.01	0.05±<0.01	0.06±<0.01	0.12±<0.01	0.25±<0.01	0.31±<0.01	4.0±<0.1	3.6±<0.1	4.8±<0.1	1.8±<0.1
		**Fold WT**	1.0	0.2	0.7	0.5	1.1	1.4	1.0	0.9	1.2	0.5
		***p*** **-values**		1.1E-27	6.4E-16	1.1E-08	8.2E-09	2.1E-14		1.2E-02	5.0E-07	3.5E-16
	**UAG**	**Recoding (%)**	0.19±<0.01	0.23±<0.01	0.17±<0.01	0.08±<0.01	0.27±<0.01	0.38±<0.01	4.8±<0.1	4.2±<0.1	5.3±<0.1	1.8±<0.1
		**Fold WT**	1.0	1.2	0.4	0.4	1.4	2.0	1.0	0.9	1.1	0.4
		***p*** **-values**		4.2E-04	4.7E-17	2.2E-15	6.7E-03	8.1E-10		8.1E-04	1.7E-03	3.6E-11
	**UGA**	**Recoding (%)**	0.55±<0.01	0.20±<0.01	0.14±<0.01	0.27±<0.01	0.55±<0.01	0.74±<0.01	8.8±<0.1	10.2±<0.1	9.4±<0.1	6.8±<0.1
		**Fold WT**	1.0	0.4	0.3	0.5	1.0	1.3	1.0	1.2	1.1	0.8
		***p*** **-values**		2.7E-16	3.7E-16	7.1E-13	1.9E-08	4.7E-22		2.4E-08	2.4E-03	2.9E-10
**Ribo. Biochemstry^d^**	**A-site Binding**	**Kd_app_ (µM)**	1.8 ±0.4					0.3 ±0.05	0.6±0.2			0.3±0.1
		**Fold WT**	1.0					0.2	1.0			0.6
	**P-site Binding**	**Kd_app_ (µM)**	0.35±0.05					0.33±0.02	2.4±0.3			2.8±0.4
		**Fold WT**	1.0					1.0	1.0			1.0
	**Petidyl Transfer**	**K_app_ (min** ^−**1**^ **)**	0.04±0.003					0.06±0.003	0.02±0.004			0.04±0.007
		**Fold WT**	1.0					1.5	1.0			1.6

Note that there are two sets of isogenic strains denoted by the heavy bars. ^a^K^+^: stable killer phenotype; K^w^: weak killer phenotype; K^−^ unable to maintain killer. ^b^Sensitivity to anisomycin or sparsomycin. NE: no effect; S: hypersensitive. ^c^All values are reported percent recoding, fold of the isogenic wild-type, and *p*-values. ^d^Ribosome biochemistry. Affinities of isogenic wild and mutant ribosomes were determined for tRNA-Phe (A-site binding), and Ac-tRNA-Phe (P-site). Apparent rates of peptidyltransfer were determined as described in Materials and methods.

### Virus propagation in rRNA modification mutants

**Figure 3:**
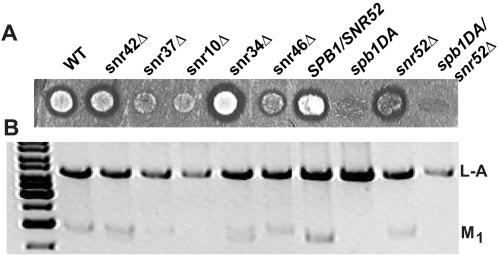
Many of the rRNA base modification mutants have M_1_ virus propagation defects. Yeast rRNA modification mutants were tested for their ability to maintain the L-A and M_1_ viruses. (A) Mutant and isogenic wild-type yeast strains were spotted onto YPAD plates, and allowed to grow at 30°C, and then replica plated to a seeded lawn of 5X47 indicator cells. Plates were incubated at room temperature for 3–5 days until a zone of inhibition was clearly visible for wild-type cells. (B) Total RNAs were extracted from mutant and isogenic wild-type yeast strains and digested with RNase A under high salt conditions. The resulting double-stranded RNA was separated on a 1% agarose gel and visualized with ethidium bromide. L-A and M_1_ dsRNAs are indicated. The image was inverted for clarity.

The yeast killer virus system composed of the dsRNA L-A helper virus and M_1_ ‘killer” satellite viruses, provides a highly sensitive assay for small defects in ribosome function. The L-A viral genome contains two overlapping ORFs, *gag* and *pol*, which encoded the structural protein and the RNA dependent RNA polymerase respectively. The two ORFs are joined by a programmed −1 ribosomal frameshift (−1 PRF) signal, and a −1 PRF event is required for synthesis a Gag-pol fusion protein. The M_1_ satellite virus dsRNA genome encodes a secreted toxin. The pre-toxin provides the infected cell with immunity to the toxin, while secretion of the mature toxin results in death of uninfected yeast cells. Alterations in −1 PRF frequencies alter the ratio of structural to enzymatic viral proteins produced for particle assembly thereby interfering with the ability of yeast to maintain the L-A helper and M_1_ satellite viruses [31]. In addition, M_1_ propagation is highly sensitive to changes in levels of free large subunits in yeast, and mutants with altered amounts of free ribosomal LSU fail to maintain the M_1_ virus [32]. To assess the effects of the mutants on virus propoagation, the L-A and M_1_ viruses were introduced into isogenic wild-type and mutant cells by cytoplasmic mixing, and cells were assayed for their abilities to maintain the killer phenotype (Fig. 3A). The wild-type strains and several of the mutants (*snr42*Δ*, snr 34*Δ*,* and *snr52*Δ**) were able to stably maintain the killer virus (K^+^). However, several of the mutants displayed defective killer phenotypes. The *snr37* *Δ* and *snr46* *Δ* mutants stably maintained the virus, but showed reductions in zones of killer activity phenotypes (K^w^). Although the killer phenotype could be initially established in *snr10* *Δ* cells, it was rapidly lost, resulting in K^−^ phenotype. The killer maintenance defect was most severe in the *spb1DA* and *spb1DA*/*snr52* *Δ* mutants where infection could not be established. Previously published data indicates altered ribosome profiles for mutants *snr10* *Δ* [18] and *spb1DA*/*snr52* *Δ* [19], which could be a contributing factor to the observed virus propagation defects. To rule out the possibility that defects in the processing or secretion of the killer toxin [32] were responsible for the observed killer phenotypes, double-stranded viral RNA was extracted from wild-type and mutant cells and visualized (Fig. 3B). The analysis revealed that M_1_ dsRNA abundance correlated with the observed killer phenotypes; i.e. M_1_ dsRNA was observed in the strains which showed the K^+^ phenotype and was absent or faint in strains that showed K^−^ or K^w^ phenotypes respectively.

### rRNA modification mutants cause allele-specific defects in translational fidelity

Assays designed to monitor various aspects of translational fidelity were employed to more precisely determine the role of rRNA modifications in translational fidelity. An *in vivo* bicistronic dual-luciferase reporter system was used to quantitatively monitor changes in −1 and +1 PRF, suppression of nonsense codons, and fidelity of aa-tRNA selection [33]. The control reporter is a yeast expression vector containing *Renilla* and firefly luciferase genes, which yields active *Renilla* and firefly luciferase proteins. Programmed −1 and +1 frameshifting test reporters were constructed by inserting a frameshift signal, L-A or Ty*1* respectively, between the *Renilla* and firefly genes such that firefly luciferase can only be produced in the event of a frameshift. *Renilla* luciferase serves as an internal control, eliminating effects due to differences in mRNA abundance, mRNA stability or translation rates between the test and control reporters. Nonsense suppression test reporters contained a stop codon (UAA, UAG, or UGA) six codons into the firefly luciferase gene, so that firefly luciferase can only be produced consequent to nonsense suppression. The misincorporation test reporters were created by mutating the firefly luciferase catalytic residue R218 from the wild-type AGA codon to either the near-cognate AGC leucine codon, or the non-cognate TCT leucine codon, so that active firefly luciferase can only synthesized when the incorrect tRNA^Arg^ is selected. Recoding efficiencies were measured for each mutant as described in the Materials and Methods. These translational fidelity data are summarized in Table 1.

The *spb1DA*/*snr52* *Δ* double mutant showed a significant increase in −1 PRF efficiency (1.5 fold of wild-type, *p* = 2.7×10^−15^), consistent with the inability of this strain to propagate the M_1_ killer virus. None of the other mutants significantly affected −1 PRF (1.2 fold or less than wild type). Similarly, none of the mutants had significant effects on +1 PRF (1.2 fold or less). The most dramatic effects of the mutants were observed in their abilities to recognize termination codons. With the exception of *snr42* *Δ*, which was hyperaccurate at UAA and UGA, but not at UAG codons, similar trends for changes in nonsense suppression were observed for all three stop codons in the other mutant strains. For example, the *snr37* *Δ*, *snr10* *Δ*, and the double mutant *spb1DA*/*snr52* *Δ* strains were hyperaccurate with respect to their ability to recognize all three termination codons, while a significant increase in general nonsense suppression was displayed in the *snr46* *Δ* mutant strain. The mutants were also assayed with regard to their ability to discriminate between sense and missense codons by using reporters described in the Supporting Information Materials and Methods S1. The *snr10* *Δ* mutant strain slightly enhanced misreading of both near and non-cognate codons (1.3 fold>wild-type), and *snr46* *Δ* showed a small increase in selection of non-cognate aa-tRNA (1.3 fold of wild type). The double mutant *spb1DA*/*snr52* *Δ* had a very significant effect on near cognate aa-tRNA misreading (1.9 fold wild-type), but did not significantly affect non-cognate aa-tRNA selection. The *spb1DA* single mutant actually exhibited a slight (0.8 fold) decrease in non-cognate aa-tRNA selection when compared to its wild-type strain with no effect on near-cognate selection events. The *snr52* *Δ* single mutant showed no change in non-cognate aa-tRNA selection events, but did display a 1.4 fold increase in near-cognate values.

### Changes in aminoacyl-tRNA binding and peptidyltransfer rates

**Figure 4:**
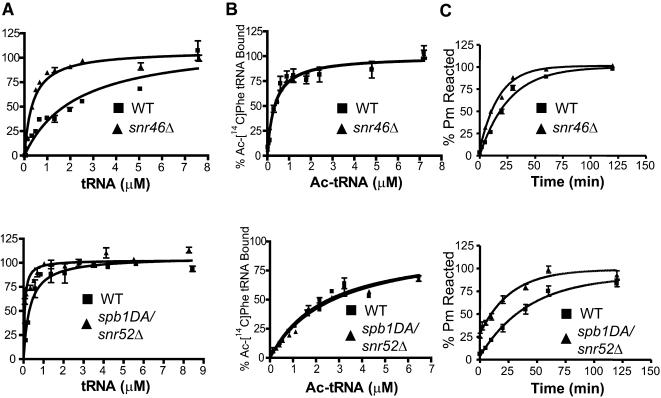
Ribosome biochemistry. Mutant strain *snr46* *Δ* and the isogenic wild-type are shown in the top row, and mutant strain *spb1DA/snr52* *Δ* and the isogenic wild-type are show in the bottom row. Error bars represent standard error for all graphs. A. [^14^C]Phe-tRNA binding to the A-site of the ribosome. One site binding curves of bound tRNA as analyzed using GraphPad Prism software. Data are reported as a percentage of the total tRNA bound. B. Ac-[^14^C]Phe-tRNA binding to the P-site of the ribosome. One site binding curves of bound tRNA as analyzed using GraphPad Prism software. Data are reported as a percentage of the total tRNA bound. C. Peptidyltransfer. Timecourse assays of peptidyltransferase activities as measured by the puromycin reaction.

Defects in translational fidelity could possibly be due to changes in tRNA binding to the ribosome or rates of peptidyltransfer. The two mutants with the most dramatic phenotypic effects, *snr46* *Δ* and *spb1DA*/*snr52* *Δ*, were selected for more detailed biochemical characterization. Ribosomes were isolated from the isogenic wild-type and mutant strains and their affinities for aa- and peptidyl-tRNA as well as peptidyltransfer rates were determined as described in the Supporting Information Materials and Methods S1. Ribosomes from both mutants had increased affinities for [^14^C]Phe-tRNA (Fig. 4A). Specifically, the Kd_app_ for *snr46* *Δ* ribosomes was 0.3 µM ±0.05, as compared to 1.8 µM ±0.4 by ribosomes isolated from the corresponding isogenic wild-type strain. Similarly, ribosomes isolated from the *spb1DA*/*snr52* *Δ* mutant had a Kd_app_ of 0.3 µM ±0.1 as compared to 0.6 µM ±0.2 for the corresponding isogenic wild-type *SPB1 SNR52* strain. In contrast, neither mutant affected binding of Ac-[^14^C]Phe-tRNA to the ribosomal P-site (Fig 4B). Peptidyltransfer rates were measured using the puromycin reaction as described in the methods. Surprisingly, both mutant strains promoted increased rates of peptidyltransfer as compared to isogenic wild-type values (Fig. 4C). Specifically, the K_app_ of *snr46* *Δ* ribosomes was 0.06 min^−1^ ±0.003 while its isogenic wild-type showed a K_app_ value of 0.04 min^−1^ ±0.003. The wild-type *SPB1 SNR52* was shown to have a K_app_ of 0.02 min^−1^ ±0.004 while the mutant strain *spb1DA*/*snr52* *Δ* showed a K_app_ of 0.04 min^−1^ ±0.007.

### The spb1DA/snr52*Δ* mutant promotes changes in key rRNA structural elements that interact with aa-tRNA

**Figure 5:**
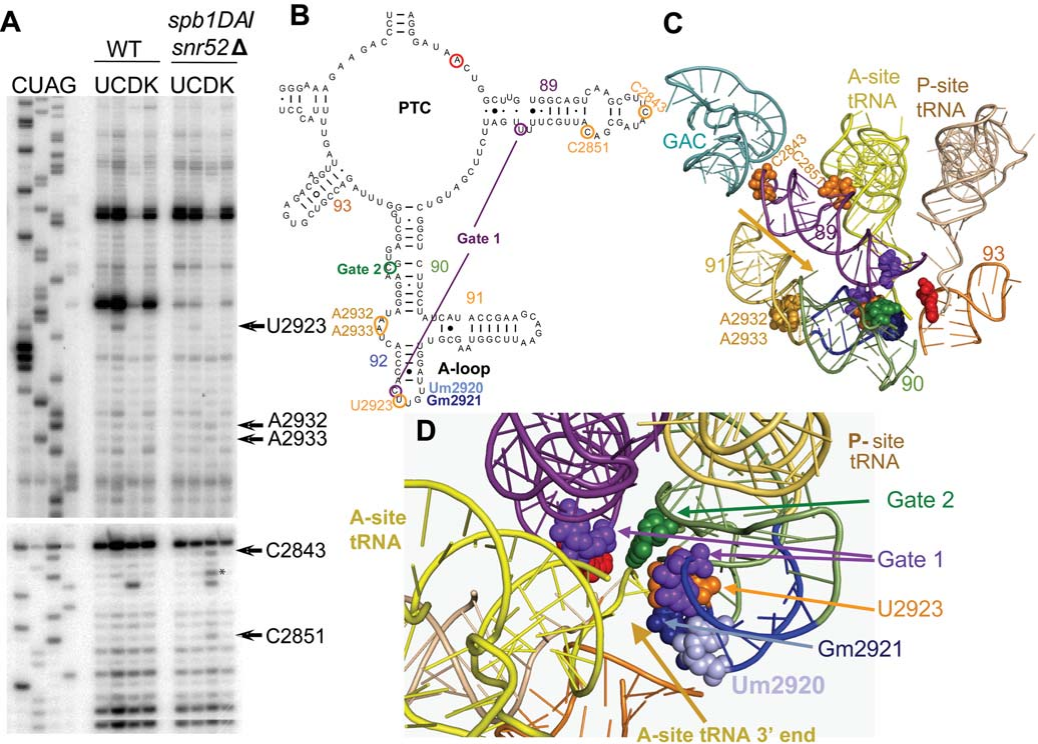
25S rRNA structure probing analysis in *spb1DA/snr52* *Δ* mutant ribosomes. (A) Puromycin treated ribosomes isolated from isogenic wild-type and *spb1DA/snr52* *Δ* mutant strains were used for *in vitro* chemical probing of the structure around the peptidyltransferase center of the ribosome, specifically helices 89-93. Reactions were performed in triplicate, representative autoradiographs are shown. U - untreated; C - CMCT; D – DMS; K – Kethoxal. Residues with changes in banding pattern labeled. Strongly modified bases at positions U2923, C2843, and C2851 are indicated, as are the more weakly deprotected A2932 and A2933. The increased intensity corresponding to U2845 (marked by *) is not DMS-specific. (B–D). Locations of residues demonstrating strong changes in protection patterns mapped to LSU rRNA structures. Um2920 and Gm2921 are indicated and color coded. Bases with altered protection patterns are circled in orange. aa-tRNA accommodation ‘gate’ bases [36] are indicated with purple (gate 1) and green (gate 2). The “catalytic” A base (equivalent to *E. coli* 23S rRNA A2451) is incicated with red. Individual LSU helices are numbered and color coded. (B) Secondary structure of yeast 25S rRNA around the PTC. (C) Three dimensional representation of rRNA bases of interest mapped onto the *E. coli* PTC [34]. Arrow represents the path the 3′ end of the aa-tRNA travels when entering the A-site. (D) Rotation and zoom in of Panel C. Shows the path into the A-site from the aa-tRNA perspective. Residues demonstrating changes in protection patterns are labeled in orange. Locations of modified bases and A-site ‘gate’ residues are labeled.

It has been speculated that the post-transcriptional rRNA modification may serve to increase the stability of the local RNA structure or decrease risk of degradation [4], [13]. With this in mind, *in vitro* rRNA structure probing was performed on the wild-type and mutant ribosomes biochemically characterized in the previous section. Mutants *snr46* *Δ* and *spb1DA*/*snr52* *Δ* and isogenic wild-type puromycin treated ribosomes were incubated with the chemically modifying agents CMCT, kethoxal and DMS *in vitro*. rRNAs were extracted and primer extension analyses performed using primers sufficient to transverse the entire PTC i.e. helices 89-93. Figure 5A shows a representative autoradiogram for the wild-type and mutant *spb1DA*/*snr52* *Δ* strains. Differences between wild-type and mutant protection patterns and their nucleotide locations are indicated. Residues C2843 and C2851 in helix 89 were deprotected from DMS, and residue U2923 in the A-loop showed increased protection from CMCT. Weaker, but consistent deprotection patterns of A2932 and A2933 were also observed. These are all mapped into the context of the 2-dimensional map of the yeast 25S rRNA (Fig. 5B), and within the atomic resolution 3-dimensional structure of the *E. coli* ribosome (Fig. 5C) [34]. The increased intensity corresponding to U2845 (marked by * in Fig. 5A) is not DMS-specific, and was not repeatable. No significant differences in protection patterns were observed between isogenic wild-type and mutant *snr46* *Δ* ribosomes (data not shown).

## Discussion

It is presently thought that rRNA base modification serves to fine tune the ribosome structure so as to optimize ribosome biogenesis and the various functions carried out by mature ribosomes. The current study has tested this hypothesis by focusing on modified bases in the A-site region of the large subunit. The data presented here provide the most detailed structure/function analysis to date, showing how minor changes in rRNA structure assure correct positioning of critical rRNA bases involved in guiding and placement of aa-tRNAs into the ribosomal A-site.

The observation of increased rates of peptidyltransfer in the *spb1DA*/*snr52* *Δ* and *snr46* *Δ* ribosomes may provide the key to understanding the function of these modified bases. Such an observation is unusual because this reaction is normally nearly instantaneous (>50/sec) [35]. To understand this, it is necessary to examine the reaction in the context of the puromyucin assay, which is approximately 3 orders of magnitude slower than peptidyltransferase assays performed with whole aa-tRNAs. One of the rate limiting aspects of the puromycin reaction is the requirement for this small molecule to diffuse into the A-site of ribosomes that are pre-loaded with peptidyl-tRNA. Thus, a simple way to increase the rate of this reaction would be to increase rates of puromycin diffusion into the active site. This could be accomplished by widening the path through which puromycin must travel in order to access the A-site. The structure probing data from *spb1DA*/*snr52* *Δ* ribosomes reveals that C2843 and C2851, two critical bases lying along the path taken by the 3′ end of the aa-tRNA during accommodation [36], are deprotected (Fig. 5A–C). This is consistent with the model of this channel being more open in this mutant. Accommodating aa-tRNA slides along the side of the helix 90 – 92 structure, and A2032 and A2933 play important roles in coordinating proper folding of this structure. Thus, the observed mild deprotection of these two bases is also consistent with the ‘open accommodation channel’ model. In addition, hyperprotection of U2923 may be due to this base collapsing into the space normally occupied by the methyl groups attached to the nearby Um2920 and Gm2921 (see Fig. 5D). This movement would drag C2922 along with it. C2922 is one of two bases that form the first “gate” through which accommodating aa-tRNA must pass [36] (shown as orange arrows in Figs. 5C and 5D). Thus, repositioning it toward the 5′ side of the A-loop would serve to open this gate, further lessening steric hindrance to puromycin.

This structural model can account for all of the other biochemical and genetic phenotypes associated with the double mutant. Opening of the aa-tRNA gates would enhance rates of diffusion of small molecules, e.g. anisomycin and sparsomycin, into the peptidyltransferase center, resulting in the hypersensitivity to these drugs observed with the *spb1DA*/*snr52* *Δ* and *snr46* *Δ* mutants respectively. This could also enhance rates of aa-tRNA accommodation, resulting in increased affinity for aa-tRNA, and increased rates of aa-tRNA misincorporation at near-cognate and termination codons. Increased −1 PRF, which involves aa-tRNA slippage could be due to loss of the interaction between C2851 and the T-stem of aa-tRNA, i.e. the aa-tRNA may not be as well fitted into the LSU, perhaps making it more prone to slip. In turn, the inability to propagate the killer virus is consistent with increased −1 PRF [37], although it should be noted that propagation of the M_1_ satellite virus is extremely sensitive to defects in the translational apparatus independent of changes in −1 PRF [38]. Importantly, +1 PRF, which only involves peptidyl-tRNA slippage [39], was not affected by these mutants, consistent with observation that none of the mutants affected binding of Ac-[^14^C]Phe to the P-site.

We speculate that the *spb1DA/snr52*Δ** double mutant displayed the most severe phenotypic defects because loss of methyl groups on 2 adjacent bases had the greatest effects on local hydration spheres, thus producing the most dramatic changes in rRNA structure. This suggests that the other mutants may also alter rRNA structure, but that these were too slight to be detected by the methods employed. However, we suggest that the effects of small structural changes are amplified through chains of biological events, thus producing the observed phenotypes. In particular, the ability of ribosomes to recognize termination codons, and the ability of cells to maintain the killer virus appear to be exquisitely sensitive indicators of subtle changes in the translational apparatus. Our conclusion is that rRNA base modification serves to fine-tune ribosome structure so as to best coordinate the structure of the molecule with its functional requirements.

There are other functionally important regions of the ribosome that contain high densities of rRNA modifications. For example, the loop of helix 69 has at least three modified residues, makes intersubunit contacts, and interacts with both A- and P-site tRNAs [2]. Functional studies in *E. coli* revealed defects in translational fidelity associated with helix 69 mutants [40]. Extensive genetic, biochemical and structural analysis of this helix would likely provide a wealth of information concerning translational fidelity and subunit association. Another interesting facet of rRNA modification is its possible role in human disease. X-linked dyskeratosis congenital (X-linked DC), marked by skin and bone marrow failure in humans, is caused by point mutations in the gene encoding the nucleolar protein dyskerin. Dyskerin is present in both the telomerase complex and in ribonucleoparticles that pseudouridylate rRNA residues. Mutations in dyskerin are associated with severe telomere dysfunction and defects in pre-rRNA processing [41]. Interestingly, cells with mutant dyskerin activity also demonstrate a defect in translation of messenger RNAs containing IRES elements [42]. It will be interesting to explore the possible relationship between rRNA modification and regulation of IRES dependent translation.

## Materials and Methods

Detailed descriptions of materials and methods are available in the Supporting Information Materials and Methods S1.

### Strains, media, and genetic methods

The *S. cerevisiae* strains used in this study are presented in Table S1. *Escherichia coli* strain DH5α was used to amplify plasmids (listed in Table S2), and *E. coli* transformations were performed using the high-efficiency transformation method [43]. Yeast cells were transformed using the alkali cation method [44]. YPAD and synthetic complete medium (H-), as well as YPG, SD, and 4.7 MB plates used for testing the killer phenotype were prepared and used as described previously [37]. Oligonucleotide primers were purchased from IDT (Coralville, IA) and are listed in Table S3. Yeast deletion strains *snr10* *Δ*, *34* *Δ*, *37* *Δ*, *42* *Δ*, *46* *Δ* and isogenic wild-type were provided by M.J. Fournier. Yeast strains s*nr52* *Δ*, s*pb1DA*, the double mutant and an isogenic wild-type were provided by G. Lutfalla. Cytoduction of the L-A and M_1_ killer virus into snoRNA knockout strains and subsequent killer virus assays were carried out as previously described [31]. Total RNAs extracted from cytoduced wild-type and snoRNA knockout strains were analyzed for the presence of the L-A and M_1_ dsRNAs, and dual luciferase assays to quantitatively monitor translational recoding in yeast were performed as previously described [33]. The latter involved use of a 0-frame control reporter in combination with −1, or +1 ribosomal frameshift, nonsense suppression, or misincorporation test reporter constructs. Recoding efficiencies and statistical analyses were performed as previously described [45]. Ten-fold dilution spot assays to monitor sensitivity to anisomycin or sparsomycin (20 µg/mL) were performed as previously described [46].

### Ribosome biochemistry

*S. cerevisiae* ribosomes were isolated, yeast aminoacyl-tRNA synthetases were purified, yeast phenylalanyl-tRNAs were aminoacylated with [^14^C]Phe, and Ac-[^14^C]tRNA was synthesized as previously described [47]. [^14^C]Phe-tRNA and Ac-[^14^C]tRNA were purified by HPLC. Peptidyltransfer assays using Ac-[^14^C]Phe-tRNA and puromycin, and equilibrium binding studies of [^14^C]Phe-tRNA binding to the ribosomal A-site, and of Ac-[^14^C]Phe-tRNA to the ribosomal P-site were carried out as previously described [47]. The data were fitted to a one site binding model using Prism Graph Pad software. Chemical protections studies of 25S rRNA employed puromycin treated ribosomes incubated with DMS (dimethyl sulfate), kethoxal or CMCT (1-cyclohexyl-3-(2-morpholinoethyl) carbodiimide metho-p-toluene), and the rRNA modifications were visualized by primer extension reactions using AMV reverse transcriptase (Roche, Mannheim, Germany) and the ^32^P-end-labeled primers shown in Table S3 were performed as previously described [48], [49].

## Supporting Information

Materials and Methods S1 (0.09 MB DOC)

Table S1 Yeast strains used in this study.
(0.04 MB DOC)

Table S2 Plasmid list.
(0.05 MB DOC)

Table S3 Oligonucleotides
(0.03 MB DOC)

## References

[1] Ban N, Nissen P, Hansen J, Moore PB, Steitz TA (2000). The complete atomic structure of the large ribosomal subunit at 2.4 A resolution.. Science.

[2] Yusupov MM, Yusupova GZ, Baucom A, Lieberman K, Earnest TN (2001). Crystal Structure of the Ribosome at 5.5 A Resolution.. Science.

[3] Samarsky DA, Fournier MJ (1999). A comprehensive database for the small nucleolar RNAs from Saccharomyces cerevisiae.. Nucleic Acids Res.

[4] Decatur WA, Fournier MJ (2002). rRNA modifications and ribosome function.. Trends Biochem Sci.

[5] Ofengand J, Bakin A, Wrzesinski J, Nurse K, Lane BG (1995). The pseudouridine residues of ribosomal RNA.. Biochem Cell Biol.

[6] Green R, Noller HF (1996). In vitro complementation analysis localizes 23S rRNA posttranscriptional modifications that are required for Escherichia coli 50S ribosomal subunit assembly and function.. RNA.

[7] Tollervey D, Lehtonen H, Jansen R, Kern H, Hurt EC (1993). Temperature-sensitive mutations demonstrate roles for yeast fibrillarin in pre-rRNA processing, pre-rRNA methylation, and ribosome assembly.. Cell.

[8] Zebarjadian Y, King T, Fournier MJ, Clarke L, Carbon J (1999). Point mutations in yeast CBF5 can abolish in vivo pseudouridylation of rRNA.. Mol Cell Biol.

[9] Lowe TM, Eddy SR (1999). A computational screen for methylation guide snoRNAs in yeast.. Science.

[10] Raychaudhuri S, Conrad J, Hall BG, Ofengand J (1998). A pseudouridine synthase required for the formation of two universally conserved pseudouridines in ribosomal RNA is essential for normal growth of Escherichia coli.. RNA.

[11] Raychaudhuri S, Niu L, Conrad J, Lane BG, Ofengand J (1999). Functional effect of deletion and mutation of the Escherichia coli ribosomal RNA and tRNA pseudouridine synthase RluA.. J Biol Chem.

[12] Charette M, Gray MW (2000). Pseudouridine in RNA: what, where, how, and why.. IUBMB Life.

[13] Helm M (2006). Post-transcriptional nucleotide modification and alternative folding of RNA.. Nucleic Acids Res.

[14] Williams DJ, Boots JL, Hall KB (2001). Thermodynamics of 2′-ribose substitutions in UUCG tetraloops.. RNA.

[15] Meroueh M, Grohar PJ, Qiu J, SantaLucia J, Scaringe SA (2000). Unique structural and stabilizing roles for the individual pseudouridine residues in the 1920 region of Escherichia coli 23S rRNA.. Nucleic Acids Res.

[16] Sumita M, Desaulniers JP, Chang YC, Chui HM, Clos L (2005). Effects of nucleotide substitution and modification on the stability and structure of helix 69 from 28S rRNA.. RNA.

[17] Gustafsson C, Persson BC (1998). Identification of the rrmA gene encoding the 23S rRNA m1G745 methyltransferase in Escherichia coli and characterization of an m1G745-deficient mutant.. J Bacteriol.

[18] King TH, Liu B, McCully RR, Fournier MJ (2003). Ribosome structure and activity are altered in cells lacking snoRNPs that form pseudouridines in the peptidyl transferase center.. Molecular Cell.

[19] Bonnerot C, Pintard L, Lutfalla G (2003). Functional redundancy of Spb1p and a snR52-dependent mechanism for the 2′-O-ribose methylation of a conserved rRNA position in yeast.. Molecular Cell.

[20] Lapeyre B, Purushothaman SK (2004). Spb1p-directed formation of Gm2922 in the ribosome catalytic center occurs at a late processing stage.. Mol Cell.

[21] Caldas T, Binet E, Bouloc P, Costa A, Desgres J (2000). The FtsJ/RrmJ heat shock protein of Escherichia coli is a 23 S ribosomal RNA methyltransferase.. J Biol Chem.

[22] Bugl H, Fauman EB, Staker BL, Zheng F, Kushner SR (2000). RNA methylation under heat shock control.. Mol Cell.

[23] Hansen TM, Baranov PV, Ivanov IP, Gesteland RF, Atkins JF (2003). Maintenance of the correct open reading frame by the ribosome.. Embo Reports.

[24] Carrasco L, Barbacid M, Vazquez D (1973). The tricodermin group of antibiotics, inhibitors of peptide bond formation by eukaryotic ribosomes.. Biochim et Biophys Acta.

[25] Grollman AP (1967). Inhibitors of protein biosynthesis. II. Mode of action of anisomycin.. J Biol Chem.

[26] Schindler D (1974). Two classes of inhibitors of peptidyltransferase activity in eukaryotes.. Nature.

[27] Hansen JL, Moore PB, Steitz TA (2003). Structures of five antibiotics bound at the peptidyl transferase center of the large ribosomal subunit.. J Mol Biol.

[28] Herner AE, Goldberg IH, Cohen LB (1969). Stabilization of N-acetylphenylalanyl transfer ribonucleic acid binding to ribosomes by sparsomycin.. Biochemistry.

[29] Jayaraman J, Goldberg IH (1968). Localization of sparsomycin action to the peptide-bond-forming step.. Biochemistry.

[30] Moazed D, Noller HF (1991). Sites of interaction of the CCA end of peptidyl-tRNA with 23S rRNA.. Proc Natl Acad Sci U S A.

[31] Dinman JD, Wickner RB (1992). Ribosomal frameshifting efficiency and Gag/Gag-pol ratio are critical for yeast M_1_ double-stranded RNA virus propagation.. J Virology.

[32] Wickner RB (1996). Double-stranded RNA viruses of *Saccharomyces cerevisiae*.. Microbiol Rev.

[33] Harger JW, Dinman JD (2003). An in vivo dual-luciferase assay system for studying translational recoding in the yeast *Saccharomyces cerevisiae*.. RNA.

[34] Schuwirth BS, Borovinskaya MA, Hau CW, Zhang W, Vila-Sanjurjo A (2005). Structures of the bacterial ribosome at 3.5 A resolution.. Science.

[35] Katunin VI, Muth GW, Strobel SA, Wintermeyer W, Rodnina MV (2002). Important contribution to catalysis of peptide bond formation by a single ionizing group within the ribosome.. Mol Cell.

[36] Sanbonmatsu KY, Joseph S, Tung CS (2005). Simulating movement of tRNA into the ribosome during decoding.. Proc Natl Acad Sci U S A.

[37] Dinman JD, Wickner RB (1994). Translational maintenance of frame: mutants of *Saccharomyces cerevisiae* with altered −1 ribosomal frameshifting efficiencies.. Genetics.

[38] Ohtake Y, Wickner RB (1995). Yeast virus propagation depends critically on free 60S ribosomal subunit concentration.. Mol Cell Biol.

[39] Belcourt MF, Farabaugh PJ (1990). Ribosomal frameshifting in the yeast retrotransposon Ty: tRNAs induce slippage on a 7 nucleotide minimal site.. Cell.

[40] Hirabayashi N, Sato NS, Suzuki T (2006). Conserved loop sequence of helix 69 in Escherichia coli 23 S rRNA is involved in A-site tRNA binding and translational fidelity.. J Biol Chem.

[41] Mochizuki Y, He J, Kulkarni S, Bessler M, Mason PJ (2004). Mouse dyskerin mutations affect accumulation of telomerase RNA and small nucleolar RNA, telomerase activity, and ribosomal RNA processing.. Proc Natl Acad Sci U S A.

[42] Yoon A, Peng G, Brandenburger Y, Zollo O, Xu W (2006). Impaired control of IRES-mediated translation in X-linked dyskeratosis congenita.. Science.

[43] Inoue H, Nojima H, Okayama H (1990). High efficiency transformation of Escherichia coli with plasmids.. Gene.

[44] Ito H, Fukuda Y, Murata K, Kimura A (1983). Transformation of intact yeast cells treated with alkali cations.. J Bacteriol.

[45] Jacobs JL, Dinman JD (2004). Systematic analysis of bicistronic reporter assay data.. Nucleic Acids Res.

[46] Meskauskas A, Dinman JD (2001). Ribosomal protein L5 helps anchor peptidyl-tRNA to the P-site in Saccharomyces cerevisiae.. RNA.

[47] Meskauskas A, Petrov AN, Dinman JD (2005). Identification of functionally important amino acids of ribosomal protein L3 by saturation mutagenesis.. Molecular & Cellular Biology.

[48] Kiparisov S, Petrov A, Meskauskas A, Sergiev PV, Dontsova OA (2005). Structural and functional analysis of 5S rRNA.. Molecular Genetics and Genomics.

[49] Rakauskaite R, Dinman JD (2006). An arc of unpaired "hinge bases" facilitates information exchange among functional centers of the ribosome.. Mol Cell Biol.

