# Supplementary material for: Optimization of Ribosome Structure and Function by rRNA Base Modification
Source: PLoS One. 2007 Jan 24;2(1):e174. doi: 10.1371/journal.pone.0000174 (PMC1766470; doi:10.1371/journal.pone.0000174)
Supplement: Materials and Methods S1 — (0.09 MB DOC) [file pone.0000174.s001.doc]

**Materials and Methods**

### *Strains, media, and genetic methods*

The *S. cerevisiae* strains used in this study are presented in Table S1. Escherichia coli strain DH5α was used to amplify plasmids (listed in Table S2), and E. coli transformations were performed using the high-efficiency transformation method [1]. Yeast cells were transformed using the alkali cation method [2]. YPAD and synthetic complete medium (H-), as well as YPG, SD, and 4.7 MB plates used for testing the killer phenotype were prepared and used as described previously [3]. Oligonucleotide primers were purchased from IDT (Coralville, IA) and are listed in Table S3. Yeast deletion strains *snr10*Δ, *34*Δ, *37*Δ, *42*Δ, *46*Δ and isogenic wild-type were provided by M.J. Fournier. Yeast strains s*nr52*Δ, s*pb1DA*, the double mutant and an isogenic wild-type were provided by G. Lutfalla.

### *Killer virus assay and viral dsRNA analyses*

Cytoduction of the L-A and M_1_ killer virus into snoRNA knockout strains and subsequent killer virus assays were carried out as previously described [4]. Briefly, viruses were introduced into [*rho*^0^] cells by cytoduction, cytoplamic mixing without mating. Cells were streaked for single colonies on selective media (-arg). Colonies were replica plated onto SD, YPG, and 4.7MB plates seeded with 5X47 killer indicator cells. Colonies were scored for growth on YPG, and the absence of growth on SD medium. Killer activity was observed after a few days at room temperature as a zone of growth inhibition around the Killer^+^ (K^+^) colonies. Total RNAs were extracted from cytoduced wild-type and snoRNA knockout strains as previously described [5]. Single stranded RNA was removed from the total RNA sample by adding 10-20μg total RNA and 0.5ng RNase A in 0.5M NaCl, 1X TE and digesting at RT for 30 min. Resulting double stranded nucleic acids were extracted once each with TE saturated phenol (pH 6.6) and chloroform, precipitated, resuspended in deionized water, and separated and visualized through an ethidium bromide stained 1% agarose TAE gel.

### *Dual Luciferase Assays*

Dual luciferase assays in yeast were performed as previously described [5]. These involve the use of a 0-frame control reporter and −1, +1 ribosomal frameshift, nonsense suppression, and misincorporation test reporter constructs. Miscoding efficiencies were calculated by determining the firefly/*Renilla* luminescence ratios from cell lysates expressing each the control and test reporters, then dividing the test ratio by the control ratio and normalizing by the wild-type. At least three readings derived from lysates derived from a minimum of three different yeast cultures were used. All assays were performed with enough replicates to achieve confidence levels of >95%, and standard errors were calculated as previously described [6]. Luminescence readings were obtained using a TD20/20 luminometer (Turner Designs Inc. Sunnyvale, CA). Reactions were carried out using the Dual-Luciferase® Reporter Assay system (Promega Corporation, Madison, WI).

### *Drug Sensitivity*

For dilution spot assays, yeast cells grown to logarithmic phase were initially diluted to 1 × 10^6^ colony forming units (CFU)/ml. Subsequent tenfold dilutions were made and 3 μl were spotted either onto rich medium on rich medium containing anisomycin or sparsomycin (10 and 20 μg/mL) and incubated at 30°C**.** Anisomycin and sparsomycin were obtained from Sigma-Aldrich, St. Louis, MO.

### *Ribosome isolation*

*S. cerevisiae* ribosomes were isolated at 4°C as previously described [7]. Cultures (I L) were grown in YPAD to O.D_595_=1.0, harvested by centrifugation, washed once with 40ml cold 0.9% KCl and frozen in liquid nitrogen. Before use, frozen cells were thawed on ice and washed again in 40ml cold 0.9% KCl. Cells were resuspended to a concentration of 1ml/2g cells in Buffer A [20 mM Tris-HCl pH 7.5 at 4^o^ C, 5 mM Mg(CH_3_COO)_2_, 50 mM KCl, 10% Glycerol, and 1mM phenylmethylsulfonyl fluoride (PMSF), 1 mM1,4-dithioerythritol (DTE) added immediately prior to use]. Cells were lysed by vigorous agitation with 0.5mm zirconia beads at 4°C, lysates were centrifuged using a Beckman micro-ultracentriguge MLS-50 rotor 25 min 20,000rpm, supernates were transferred to a 5ml pollyallomer tube containing a 1 ml cushion of cold Buffer C [20 mM Tris-HCl pH 7.5 at +4^o^ C, 5 mM Mg(CH_3_COO)_2_, 50 mM KCl, 25% Glycerol, and 0.1 mM PMSF, 0.1 mM DTE], and centrifuged 2 hours 20min at 50,000 rpm. Supernates were was discarded and fines were gently washed away from ribosome pellets with 1ml Buffer C. Ribosomes were resuspended in 100 to 300 μL cold Buffer C. Samples were centrifuged in a microfuge for 1 min 12000 rpm 4°C, supernates were transferred to new tubes, and ribosomes were aliquoted and stored at -80°C. Concentrations were determined using optical density (1 OD_260_ = 20 pmol).

### *Purification of aminoacyl-tRNA synthetases*

Aminoacyl-tRNA synthetases were purified as previously described [7]. Two pounds of frozen cake yeast (George R. Ruhl & Son, Inc., Hanover, MD) were placed in 500 ml of buffer A [0.2 M Tris-base, 0.3 M NH_4_Cl, 20 mM MgSO_4_, 1 mM EDTA, 0.15 M dextrose] and allowed to thaw and ferment overnight. Cells were disrupted by three passages through an ice-cooled Microfluidaser at ~18,000 lb/in^2^, cell debris was removed by centrifugation at 4 °C in a Beckman JLA rotor at 10,000 rpm for 30 minutes, and 800 ml of supernatant was obtained. Fines and nucleic acids were precipitated by addition of polyethylenimine (1.73 g/lb of cells, equivalent to 4.32 g/liter of lysate) over a period of 5 minutes with slow stirring. Precipitates were removed by centrifugation at 4 °C using a GSA rotor at 9,000 rpm for 40 minutes. Proteins in the supernatant were precipitated by addition of 472 g of ammonium sulfate per liter of extract (70 % saturation), and precipitates were collected by centrifugation in a GSA rotor at 12,000 rpm for 45 minutes at room temperature. The pellet from this step was suspended in 43.75 ml of buffer C [30 mM potassium phosphate, pH 7.2, 1 mM EDTA, 1 mM DTE, 0.01 mM PMSF] per 100 g of pellet and subsequently dialyzed in 2 liters of buffer C overnight with two changes of buffer. The extract then was clarified by centrifugation in a GSA rotor at 12,000 rpm for 45 minutes at 4 °C. The supernatant was diluted 2.5 times with buffer C and fractionated through a Sephadex CM50 column equilibrated with buffer C. The column was washed with buffer D [30 mM potassium phosphate, pH 7.2, 1 mM EDTA, 0.01 mM PMSF, 10 % glycerol] with 50 mM KCl. The proteins were eluted from the column using a series of step gradients composed of buffer D containing 150 mM, 300 mM, and 500 mM KCl. The material eluted by buffer D with 150 mM KCl contains phenylalanyl-tRNA synthetase activity. Proteins were precipitated by addition of 472 g/liter of ammonium sulfate, and pellets were suspended in buffer D containing 50 mM KCl. Extracts were dialyzed against 1 liter with two changes of buffer D50 for 10 h, after which they were clarified by centrifugation in a GSA rotor at 12,000 rpm for 45 minutes at 4 °C. The obtained preparations of aa-tRNA synthetases were aliquoted and flash frozen in liquid nitrogen.

### *Synthesis of aminoacyl-tRNA and acetylated aminoacyl-tRNA*

Yeast phenylalanyl-tRNAs were aminoacylated as previously described [7]. The reaction mix (5 ml) contained 300 mM Tris-HCl, pH 7.6, 100 mM KCl, 20 mM MgCl_2_, 0.4 mM ATP, 40 µM [^14^C]Phe [496 mCi/mmole], plus 5 mg of tRNA-Phe and 475 µl of aminoacyl-tRNA synthetases (D150) purified as described above. Reaction mixtures were incubated for 30 minutes at 30 °C, and proteins were removed by extraction with acid-phenol-chloroform. [^14^C]Phe-tRNA was separated from uncharged tRNA and free [^14^C]Phe by high-performance liquid chromatography (HPLC) as previously described [8] with the following modifications. Samples were loaded onto a 4.6x250 mm JT Baker wide-pore butyl column equilibrated with buffer A [20 mM NH_4_(CH_3_COO), 10 mM MgCl_2_, 400 mM NaCl; pH 5.0] at 1 ml/min. The column was washed with 10 ml of buffer A, creating conditions under which free phenylalanine and aminoacyladenylate are eluted from the column. Uncharged tRNAs were eluted by isocratic elution with 19 ml at 15 % of buffer B [20 mM NH_4_(CH_3_COOH), 10 mM MgCl_2_, 400 mM NaCl, 60 % methanol; pH 5.0). [^14^C]Phe-tRNA was eluted using a step gradient to 100 % of buffer B. Elution of aminoacyl-tRNA was monitored by OD_260/280_ readings, and [^14^C]Phe-tRNA peak and concentrations were determined by scintillation counting. The presence of aminoacyl-tRNA in the eluted material was confirmed by gel filtration through G-25 spin columns and by nonenzymatic hydrolysis of ester bonds at basic pH [9]. Ac-[^14^C]tRNA was obtained in a similar manner. Yeast phenylalanyl tRNA was charged with [^14^C]Phe as above and extracted with phenol. The [^14^C]Phe-tRNA was acetylated by addition of 64 µl of acetic anhydride at 15 minutes intervals for 1 h on ice [8]. The reaction mix was clarified by centrifugation at 15,000 rpm for 3 minutes, and Ac-[^14^C]Phe-tRNA was purified by HPLC as described above.

### *Characterization of peptidyltransferase activity*

Peptidyltransfer assays were performed essentially as previously described [7]. The protocol was carried out on ice or at 4°C at all times unless otherwise noted. Briefly, complex C [80S ribosomes, Ac-Phe-tRNA, poly(U)] was formed by incubating 120 pmol ribosomes, 0.4 mg/ml poly(U), 0.4 mM GTP, and 100 pmol Ac- [^14^C]Phe-tRNA in 200 μL of binding buffer P [80 mM Tris-HCl, pH 7.4, 11 mM magnesium acetate, 160 mM ammonium chloride, 6 mM β-mercaptoethanol, and 2 mM spermidine] for 20 min at 30°C. The complex was filtered through a Millipore HA filter, and washed with binding buffer. Complex C was extracted off the filter disk by gently shaking in binding buffer containing 0.05% of Zwittergent 3-12 for 30min at 4°C. Complex C extract was pre-incubated at 30°C for 5 min to activate ribosomes. 2mM puromycin was added to complex C in 100 μL of binding buffer P to begin the reaction. Time points were taken at 0, 2, 5, 10, 20, 30, 60, 120 min by removing 100 μL aliquots and terminating the reactions by addition of 100 μL of 1.0 N NaOH. Reaction products were extracted with 0.5 ml of ethyl acetate, and radioactivity was determined by scintillation counting. Control values of reaction mixture without puromycin and of extracted complex C were obtained in each experiment. The data were analyzed using Prism Graph Pad software and was fit to give the value of *K*_app_, the apparent rate constant of the entire reaction at a given concentration of puromycin using the equation Y=-Ae^(-kt)+C where Y is the normalized counts per minute (CPM), C is the final value of the normalized CPM, and t is the time in minutes.

### *tRNA binding activity*

Aminoacyl-tRNA binding to the A-site of the ribosome was carried out as previously described [7]. Briefly, a reaction mixture of 12-25 pmol ribosomes, 0.4 mg/ml poly (U), 0.4 mM GTP, and in 50 μl of binding buffer A [80 mM Tris-HCl, pH 7.4, 11 mM magnesium acetate, 160 mM ammonium chloride, 6 mM β-mercaptoethanol, and 2 mM spermidine] was preincubated with uncharged tRNA (4:1 tRNA/ribosomes) at 30°C for 15 min to occupy ribosomal P- and E-sites by uncharged tRNA. The mixture is added to increasing amounts of [^14^C]Phe-tRNA (4 to 264 pmol) and incubated at 30°C for an additional 15 min to allow formation of [14C]Phe-tRNA–80S–poly (U) complexes. Aliquots were then applied to nitrocellulose membranes, washed, and the resulting radioactivity of the membrane was measured by scintillation counting. Background levels of radioactivity were determined using a blank sample and subtracted from the test samples. The data were analyzed using Prism Graph Pad software and was fit to a nonlinear regression one site binding curve (Y=B_max_*X/(K_d_ + X)) where Y is normalized CPM values, and X is tRNA concentration in μM.

Acetyl-aminoacyl-tRNA binding to the P-site of the ribosome was carried out as similar to the A-site binding protocol with the following modifications. Briefly, purified ribosomes in Buffer C were thawed on ice and treated with 1mM puromycin and 1mM GTP (adjust PMSF and DTE to 1mM) and incubated at 30°C for 30 min to remove aminoacyl-tRNAs. A reaction mixture of 12-25 pmol treated ribosomes, 0.4 mg/ml poly(U), and 50μl binding buffer P [80 mM Tris-HCl, pH 7.4, 11 mM magnesium acetate, 160 mM ammonium chloride, 6 mM β-mercaptoethanol, and 2 mM spermidine] is added to increasing amounts of Ac-[^14^C]Phe-tRNA (4 to 264 pmol) and incubated at 30°C for 15 min to allow formation of [14C]Phe-tRNA–80S–poly(U) complexes. The protocol then proceeds as described above for aminoacyl-tRNA binding reactions.

### *Structure Probing on Ribosomes in vitro*

Purified ribosomes in Buffer C were thawed on ice and treated with 1mM puromycin and 1mM GTP (adjust PMSF and DTE to 1mM) and incubated at 30°C for 30 min to remove aminoacyl-tRNAs. Puromycin treated ribosomes were incubated with DMS (dimethyl sulfate), Kethoxal or CMCT (1-cyclohexyl-3-(2-morpholinoethyl) carbodiimide metho-p-toluene) as previously described [10]. Treated rRNA was extracted and slow-cool annealed in annealing buffer (250mM TrisCl pH 8.3, 200mM KCl) to ^32^P-end-labeled primers (Table 4) which are used to investigate the ribosomal peptidyl-transferase center and A-loop. Primer extension and RNA sequencing were both performed using AMV reverse transcriptase (Roche, Mannheim, Germany) at 42°C for 30 min. Primer extensions were performed using 4μM dNTPs. Reaction products were separated through a 10% urea-polyacrylamide gel and visualized using a BioRad phosphoimager.

References

1. Inoue H, Nojima H, Okayama H (1990) High efficiency transformation of Escherichia coli with plasmids. Gene 96: 23-28.

2. Ito H, Fukuda Y, Murata K, Kimura A (1983) Transformation of intact yeast cells treated with alkali cations. J Bacteriol 153: 163-168.

3. Dinman JD, Wickner RB (1994) Translational maintenance of frame: mutants of *Saccharomyces cerevisiae* with altered -1 ribosomal frameshifting efficiencies. Genetics 136: 75-86.

4. Dinman JD, Wickner RB (1992) Ribosomal frameshifting efficiency and Gag/Gag-pol ratio are critical for yeast M_1_ double-stranded RNA virus propagation. J Virology 66: 3669-3676.

5. Harger JW, Dinman JD (2003) An in vivo dual-luciferase assay system for studying translational recoding in the yeast *Saccharomyces cerevisiae*. RNA 9: 1019-1024.

6. Jacobs JL, Dinman JD (2004) Systematic analysis of bicistronic reporter assay data. Nucleic Acids Res 32: e160-e170.

7. Meskauskas A, Petrov AN, Dinman JD (2005) Identification of functionally important amino acids of ribosomal protein L3 by saturation mutagenesis. Molecular & Cellular Biology 25: 10863-10874.

8. Triana-Alonso FJ, Spahn CM, Burkhardt N, Rohrdanz B, Nierhaus KH (2000) Experimental prerequisites for determination of tRNA binding to ribosomes from Escherichia coli. Methods Enzymol 317: 261-276.

9. Kaneko I, Doi RH (1966) Alteration of valyl-sRNA during sporulation of bacillus subtilis. Proc Natl Acad Sci U S A 55: 564-571.

10. Kiparisov S, Petrov A, Meskauskas A, Sergiev PV, Dontsova OA, Dinman JD (2005) Structural and functional analysis of 5S rRNA. Molecular Genetics and Genomics 27: 235-247.
