# Supplementary material for: Optimization of Ribosome Structure and Function by rRNA Base Modification
Source: PLoS One. 2007 Jan 24;2(1):e174. doi: 10.1371/journal.pone.0000174 (PMC1766470; doi:10.1371/journal.pone.0000174)
Supplement: Table S1 — Yeast strains used in this study. (0.04 MB DOC) [file pone.0000174.s002.doc]

| **Strain** | **Description** | **Source** |
| --- | --- | --- |
| 5X47 | *MAT*a/*MAT*α *his1*/+ *trp1*/+ *ura3*/+ K^–^; Standard diploid killer tester | Dinman Lab |
| JD758 | *MAT***a** *kar1-1 arg1* [L-AHN M_1_] | Dinman Lab |
| JD759 | MATα *kar1-1 arg1* [L-A HN M_1_] | Dinman Lab |
| JD932D | *MAT***a** *ade2-1 trp1-1 ura3-1 leu2-3,112 his3-11,15 can1-100* [L-AHN M_1_] | Dinman Lab |
| 1187 | *MATα ade2-101 trp1-Δ101 ura3-52 leu2-3, 112 his3Δ200* | M.J. Fournier |
| 1188 | *MATα ade2-101 trp1-Δ101 ura3-52 leu2-3, 112 his3Δ200 snr42::HIS3* | M.J. Fournier |
| 1189 | *MATα ade2-101 trp1-Δ101 ura3-52 leu2-3, 112 his3Δ200 snr37::URA3* | M.J. Fournier |
| 1190 | *MATa ade2-101 trp1-Δ101 ura3-52 leu2-3, 112 his3Δ200 snr10::LEU2* | M.J. Fournier |
| 1191 | *MATα ade2-101 trp1-Δ101 ura3-52 leu2-3, 112 his3Δ200 snr34::LEU2* | M.J. Fournier |
| 1192 | *MATα ade2-101 trp1-Δ101 ura3-52 leu2-3, 112 his3Δ200 snr46::HIS3* | M.J. Fournier |
| 1316 | *MATa ade2-1 his3-11 leu2-3 112 trpΔ ura3-1 can1-100 spb1Δ::TRP1 pSEY18-SPB1-OR19(CEN, URA3,Spb1)* | G. Lutfalla |
| 1317 | *MATa ade2-1 his3-11 leu2-3 112 trpΔ ura3-1 can1-100 spb1Δ::TRP1 p(CEN, LEU2,HASpb1DA)* | G. Lutfalla |
| 1318 | *MATα ade2-1 his3-11 leu2-3 112 trpΔ ura3-1 can1-100 snR52Δ::TRP1* | G. Lutfalla |
| 1319 | *MATa ade2-1 his3-11 leu2-3 112 trpΔ ura3-1 can1-100 spb1Δ::TRP1 snR52Δ::TRP1 p(CEN, LEU2,HASpb1DA)* | G. Lutfalla |

# Table S1. Yeast strains used in this study.
