# Supplementary material for: Optimization of Ribosome Structure and Function by rRNA Base Modification
Source: PLoS One. 2007 Jan 24;2(1):e174. doi: 10.1371/journal.pone.0000174 (PMC1766470; doi:10.1371/journal.pone.0000174)
Supplement: Table S2 — Plasmid list. (0.05 MB DOC) [file pone.0000174.s003.doc]

| **Strain** | **Description** |
| --- | --- |
| pJD375 | 0-frame control dual luciferase reporter test (DLR). Harbors a *URA3* selectable marker. |
| pJD376 | -1 PRF test DLR. Harbors a *URA3* selectable marker. |
| pJD 377 | +1 PRF test DLR. Harbors a *URA3* selectable marker. |
| pJD 419 | 0-frame control dual luciferase reporter test (DLR). DLR cassette identical to that in pJD375. Harbors a *LEU2* selectable marker. |
| pJD 420 | -1 PRF test DLR. DLR cassette identical to that in pJD376. Harbors a *LEU2* selectable marker. |
| pJD 421 | +1 PRF test DLR. DLR cassette identical to that in pJD377. Harbors a *LEU2* selectable marker. |
| pJD431 | Nonsense suppression test DLR . Contain the UAA premature termination codon. Harbors a *URA3* selectable marker. |
| pJD 432 | Nonsense suppression test DLR . Contain the UAG premature termination codon. Harbors a *URA3* selectable marker. |
| pJD433 | Nonsense suppression test DLR . Contain the UGA premature termination codon. Harbors a *URA3* selectable marker. |
| pJD633 | 0-frame control dual luciferase reporter test (DLR). DLR cassette identical to that in pJD375. Harbors a *TRP1* selectable marker. |
| pJD634 | -1 PRF test DLR. DLR cassette identical to that in pJD376. Harbors a *TRP1* selectable marker. |
| pJD635 | +1 PRF test DLR. DLR cassette identical to that in pJD377. Harbors a *TRP1* selectable marker. |
| pJD642 | Non-cognate tRNA misincorporation test DLR. Harbors a *URA3* selectable marker. |
| pJD643 | Near-cognate tRNA misincorporation test DLR. Harbors a *URA3* selectable marker. |
| pJD676 | Non-cognate tRNA misincorporation test DLR. DLR cassette identical to that of pJD643. Harbors a *TRP1* selectable marker. |
| pJD677 | Near-cognate tRNA misincorporation test DLR. DLR cassette identical to that of pJD644. Harbors a *TRP1* selectable marker. |
| pJD699 | Non-cognate tRNA misincorporation test DLR. Harbors a *URA3* selectable marker. |
| pJD700 | Near-cognate tRNA misincorporation test DLR. DLR cassette identical to that of pJD644. Harbors a *TRP1* selectable marker. |
| pJD702 | Non-cognate tRNA misincorporation test DLR. Harbors a *URA3* selectable marker. |
| pJD703 | Nonsense suppression test DLR. Contain the UAG premature termination codon. DLR cassette identical to that of pJD43. Harbors a *LEU2* selectable marker. |
| pJD704 | Nonsense suppression test DLR. Contain the UGA premature termination codon. DLR cassette identical to that of pJD433. Harbors a  *LEU2* selectable marker. |

**Table S2. Plasmid List**
