# Supplementary material for: Optimization of Ribosome Structure and Function by rRNA Base Modification
Source: PLoS One. 2007 Jan 24;2(1):e174. doi: 10.1371/journal.pone.0000174 (PMC1766470; doi:10.1371/journal.pone.0000174)
Supplement: Table S3 — Oligonucleotides (0.03 MB DOC) [file pone.0000174.s004.doc]

| **Name and Description** | **Sequence**  **5’ – 3’** |
| --- | --- |
| Universal reverse sequencing primer | TTCACACAGGAAACAG |
| Universal forward sequencing primer | GTAAAACGACGGCCAGT |
| 25-6  Ribosome structure probing helices 93-89 | AACCTGTCTCACGACGG |
| 25-10  Ribosome structure probing h89 | GGTATGATAGGAAGAGC |

**Table S3: Oligonucleotides**
